# *AoMYB114* transcription factor regulates anthocyanin biosynthesis in the epidermis of tender asparagus stems

**DOI:** 10.3389/fpls.2025.1531574

**Published:** 2025-02-18

**Authors:** Yanwen Li, Mengyao Li, Zheng Guo, Junting Liu, Peiran Chen, Wei Lu, Chengyao Jiang, Jiachang Xiao, Fengyun Lei, Yangxia Zheng

**Affiliations:** ^1^ College of Horticulture, Sichuan Agricultural University, Chengdu, China; ^2^ Agricultural Equipment Research Institute, Chengdu Academy of Agricultural and Forest Sciences, Chengdu, China

**Keywords:** *Asparagus officinalis* L., epidermis color, transcriptome, anthocyanin, MYB

## Abstract

**Introduction:**

Asparagus is a valuable vegetable, and its edible part is a tender stem. The color of the tender stem epidermis is an important trait. In particular, purple asparagus is rich in anthocyanins. However, the molecular mechanisms underlying anthocyanin accumulation in purple asparagus remains unclear.

**Methods:**

The white variety ‘Jinguan’ (JG), the green variety ‘Fengdao 2’ (FD), and the purple variety ‘Jingzilu 2’ (JZ) were compared using physiological and transcriptomic analysis. High-performance liquid chromatography and real-time quantitative polymerase chain reaction were employed to detect anthocyanins and validate gene expression.

**Results:**

Cyanidin 3-glucoside and cyanidin 3-rutinoside were detected as the main anthocyanins in JZ. Transcriptome data demonstrated that 4,694 and 9,427 differentially expressed genes (DEGs) were detected in the JZ versus FD and JZ versus JG control groups, respectively. These DEGs were significantly enriched in pathways associated with anthocyanin accumulation, including phenylalanine metabolism, phenylpropanoid biosynthesis, and flavonoid biosynthesis. A total of 29 structural genes related to anthocyanin biosynthesis were identified. The expression of these structural genes was higher in JZ than in FD and JG, thereby activating the anthocyanin biosynthesis pathway. Additionally, a candidate gene, *AoMYB114*, was identified based on transcriptomic data. The expression of *AoMYB114* was associated with anthocyanin accumulation in different tissues. Further research found that overexpression of *AoMYB114* activated the anthocyanin biosynthesis pathway. It promoted leaf pigment accumulation in transgenic *Arabidopsis*.

**Discussion:**

These findings demonstrate that *AoMYB114* positively regulated anthocyanin biosynthesis. This study elucidates the molecular mechanism underlying purple coloration in asparagus. It provides important insights for improving asparagus quality and for breeding high-anthocyanin varieties.

## 1 Introduction

Plants exhibit various colors due to pigmented substances within their cells. Pigments absorb some of the sun’s rays, while other rays are perceived by vision through reflection. Plant pigments are divided into four main categories: chlorophylls, carotenoids, flavonoids, and alkaloids. Chlorophylls are primarily green, and carotenoids are orange-red. Anthocyanins from the flavonoid group are the most significant color-developing substances (Smeriglio et al., 2016). It is widely present in plants and influences the color and quality of flowers, fruits, and vegetables. Many anthocyanins exist in nature, but their basic body structures remain the same. The primary differences are the type and number of substituent groups, binding sites, and glycosidic ligands (Tanaka et al., 2008). The commonly available anthocyanins currently include six groups: pelargonidin, cyanidin, delphinidin, peonidin, petunidin, and malvidin (Tanaka et al., 2008; Kong et al., 2003). Plant flowers, leaves, and fruit color differ depending on the anthocyanin substrate and chemical modification. Its accumulation also plays a role in many biological processes, including ultraviolet radiation absorption (Quina et al., 2009), insect attraction for pollination (Ogutcen et al., 2020), participation in biotic stresses, and responses to abiotic stresses, including low temperatures, drought, and high salinity (Kovinich et al., 2015, 2014). In addition, anthocyanins have the function of scavenging free radicals and improving the antioxidant capacity of organisms (Cappellini et al., 2021). Due to their natural activity reducing oxidative stress in the body, they also have a positive role in maintaining cardiovascular health in humans (Krga and Milenkovic, 2019).

The anthocyanin biosynthesis pathway is relatively conserved and has been characterized in many plants (Tanaka et al., 2008; Holton and Cornish, 1995). The pathway is derived from the phenylalanine pathway (Sunil and Shetty, 2022) and involves in several key enzymes. This pathway primarily includes early biosynthetic enzymes, including chalcone synthase (CHS), chalcone isomerase (CHI), and flavonoid 3-hydroxylase (F3H), and late biosynthetic enzymes, such as dihydroflavonol 4-reductase (DFR) and anthocyanin synthase (ANS) (Colanero et al., 2020; Tanaka and Brugliera, 2013). Structural genes encoding these enzymes have been identified and correlated in numerous species, including petunia (De Paoli et al., 2009), mulberry (Chao et al., 2021), and eggplant (Wu et al., 2020). The specific functions of these proteins have been verified.

Transcription factors, microRNAs and long non-coding RNAs regulate the expression of structural genes in the anthocyanin biosynthetic pathway (Yang et al., 2019). Myeloblastosis (MYB), basic helix-loop-helix (bHLH), and W40 repeat domain proteins (WD40) are major transcription factors. These three transcription factors can form MYB-bHLH-WD40 complex (MBW) via protein interactions (Xu et al., 2015). R2R3-MYB transcription factors are critical for activating the MBW complex (Liu et al., 2018). Most can positively regulate anthocyanin accumulation, while a few can negatively regulate it. This has been verified in various liliaceous plants. For example, *LhMYB12* expression in an Asian hybrid lily (*Lilium* spp.) corresponds to anthocyanin pigmentation in the perianth, filaments, and patterns. Simultaneously, *LhMYB6* regulates anthocyanin spot development in the perianth. *LhMYB12* and *LhMYB6* positively regulate anthocyanin biosynthesis (Yamagishi et al., 2010). *MaAN2*, an anthocyanin-related R2R3-MYB gene, was identified from transcriptome data of grape hyacinth (*Muscari armeniacum*). Anthocyanin accumulation and *MaAN2* expression were also positively correlated. They also play a positive role in anthocyanin biosynthesis (Chen et al., 2017). In addition, *TgMYB4*, an R2R3-MYB transcription factor, has been isolated from the petals of tulip (*Tulipa gesneriana* L.). Tobacco plants overexpressing *TgMYB4* exhibited white or light pink petals with less anthocyanin accumulation than control plants. Therefore, *TgMYB4* inhibits the regulation of anthocyanin biosynthesis during anthocyanin deposition (Hu et al., 2024).

Asparagus (*Asparagus officinalis* L.) is a perennial herb belonging to the genus Asparagus in the Liliaceae and is native to the Mediterranean coast. It is renowned for its nutritional value and unique flavor. It is also known as the king of vegetables and one of the world’s top 10 famous dishes (He et al., 2024; Xiao et al., 2024). Asparagus is classified into three categories: white, green, and purple, depending on the color of the tender stem. Purple Asparagus is rich in anthocyanins and possesses stronger antioxidant properties than white and green asparagus (Olas, 2024). It also has various biological activities, including anti-inflammatory and antibacterial. Consequently, it plays an essential role in human health (Krga and Milenkovic, 2019; Wang and Ng, 2001). Anthocyanin biosynthesis and regulation have been intensively studied in many plants. However, few studies have reported anthocyanins in tender asparagus stems. Only the environmental regulation of anthocyanin biosynthesis is involved. Liang (Liang et al., 2022) indicated that high temperatures inhibit anthocyanin accumulation in purple asparagus. Dong (Dong et al., 2019) indicated that anthocyanin accumulation in asparagus is light-dependent. Overall, it is difficult for previous studies to comprehensively explore the molecular mechanisms involved in purple asparagus stem formation. Particularly, the cloning, isolation, and characterization of transcription factors related to controlling epidermal purple traits have not been reported. In this study, the transcriptional differences in different epidermal colors were investigated. The functions of MYB transcription factors involved in anthocyanin biosynthesis were verified. This laid an important foundation for a further comprehensive and in-depth interpretation of the color mechanism of purple asparagus tender stems.

## 2 Materials and methods

### 2.1 Plant materials

In this study, three-year-old asparagus of white variety ‘Jinguan’ (JG), green variety ‘Fengdao 2’ (FD), and purple variety ‘Jingzilu 2’ (JZ) were selected. Three varieties were cultivated in the asparagus base of the Enyang District, Bazhong City, Sichuan Province (106.56°E, 31.65°N) at an altitude of 580 m. Robust tender asparagus stems with essentially uniform growth conditions and free from pests and diseases were selected as samples in June 2022. Samples were obtained by cutting the epidermis downward from the tip. Three biological replicates were used for each variety. All samples were immediately frozen in liquid nitrogen and stored in an ultra-low-temperature refrigerator at −80 °C.

### 2.2 Determination of pigment content and color parameters

Chlorophyll and carotenoid levels in the asparagus epidermis were measured using spectrophotometry. The total anthocyanin content in the asparagus epidermis was measured using the pH differential method. Anthocyanin components were determined using high-performance liquid chromatography (HPLC), as described by Fan et al. (2023), with minor modifications. An Agilent 1260 liquid chromatograph equipped with a ComatexC18 column (250 × 4.6 mm, 5 μm) was used for determination. Mobile phase A was a pure acetonitrile solution, whereas mobile phase B was a 1.6% aqueous formic acid solution. The gradient elution procedure is presented in **Supplementary Table S1**. Standard curves were generated using pure standard samples of cornflowerin-3-O-glucoside and cornflowerin-3-O-rutinoside (purity ≥ 98%, Solarbio Technology, Beijing, China). The color of the asparagus stems was measured using a CR-400 colorimeter (Konica Minolta, Tokyo, Japan).

### 2.3 Transcriptome sequencing and data analysis

RNA was extracted from three different colors of the asparagus stem epidermis using the Trizol method. The quality of the asparagus RNA was controlled using an Agilent Bioanalyzer 2100 (Agilent Technologies, Santa Clara, CA, USA). A cDNA library was constructed and sequenced using the Illumina HiSeq 6000 platform. Transcriptome sequencing was performed by Novogene Biotech (Beijing, China). Reads with adapter reads containing N (N indicates that base information cannot be determined) and low-quality reads (reads with Qphred ≤ 20 base number accounting for > 50% of the entire read length) were eliminated to ensure the quality and reliability of the data analysis. High-quality clean reads were obtained.

Gene expression levels were calculated using FPKM (one thousand base transcript fragments per million drawn). Differentially expressed genes (DEGs) between colors were analyzed using DESeq2 software (1.20.0, Bioconductor, Boston, MA, USA). The screening criteria were |log2(Fold Change)| ≥ 1 and padj ≤ 0.05 (Anders and Huber, 2010). Gene Ontology (GO) and Kyoto encyclopedia of genes and genomes (KEGG) enrichment analyses of DEGs were performed using the cluster Profiler software (version 3.8.1) to elucidate the signaling pathways involved in the differential genes. The predicted transcription factors were screened and categorized using the PlantTFDB database (http://planttfdb.gao-lab.org/).

### 2.4 Verification of gene expression levels

Gene-specific primers were designed using Primer Premier 6 (**Supplementary Table S2**). RNA was extracted using the Plant Total RNA Isolation Kit (Foregene, Chengdu, China), and cDNA was reverse transcribed from the extracted RNA using Goldenstar™ RT6 cDNA Synthesis Mix RNasin (Tsingke, Beijing, China). Real-time quantitative polymerase chain reaction (RT-qPCR) was performed using a fluorescence RT-qPCR system Bio-Rad CFX96TM and SYBR Green I (Tsingke, Beijing, China). The data were analyzed using the 2^−ΔΔCt^ method (Livak and Schmittgen, 2001). *AoUbiq*, a ubiquitin long-tail gene (Ma et al., 2023), was used as a reference gene to normalize RT-qPCR results for asparagus.

### 2.5 Cloning of *AoMYB114*, overexpression vector construction, and *Arabidopsis* genetic transformation

*AoMYB114*, a transcription factor, was selected based on the transcriptome sequencing annotation information and gene expression of tender asparagus stems. The cDNA of JZ was amplified using a template. The sequence of cloning primers is presented in **Supplementary Table S2**. The fragment was cloned to a pUCm-T vector (Sangon Biotech, Shanghai, China) and transformed into *Escherichia coli* DH5α (Tsingke, Beijing, China). The plasmid was extracted, identified using PCR, and sequenced by Tsingke (Beijing, China). Recombinant primers were designed according to the gene and vector sequences (**Supplementary Table S2**). BamHI and SacI restriction sites were selected, and the *AoMYB114* gene was amplified using recombinant primers and recombined into the pCAMBIA-1301 vector. The recombinant plasmid transformed *Escherichia coli* DH5α, and the positive bacterial liquid detected by PCR was sent to Tsingke (Beijing, China) for sequencing.

Genetic transformation of *Arabidopsis thaliana* was conducted using the binary expression vector pCAMBIA-1301. The vector contained the GUS reporter gene and the Kan resistance gene. The recombinant plasmid, pCAMBIA1301-AoMYB114, was transferred into Agrobacterium GV3101. Genetic transformation of *Arabidopsis thaliana* was performed using the floral dip method (Zhang et al., 2006). Transgenic *Arabidopsis* plants were screened on 1/2 MS solid medium containing hygromycin. The transgenic strains were further verified using PCR amplification and GUS staining.

### 2.6 Determination of total anthocyanin content and components in transgenic *Arabidopsis thaliana* and expression analysis of genes related to anthocyanin biosynthesis

The methods of total anthocyanin determination and gene quantitative analysis of *Arabidopsis thaliana* were the same as above. The RT-qPCR results for *Arabidopsis thaliana* were normalized using *AtActin2* as a reference gene. The RT-qPCR procedure and primers for *Arabidopsis thaliana* have been previously described (Xu et al., 2017). Primer sequences are presented in **Supplementary Table S2**.

## 3 Results

### 3.1 Pigmentation analysis of different asparagus

**Figure 1:**
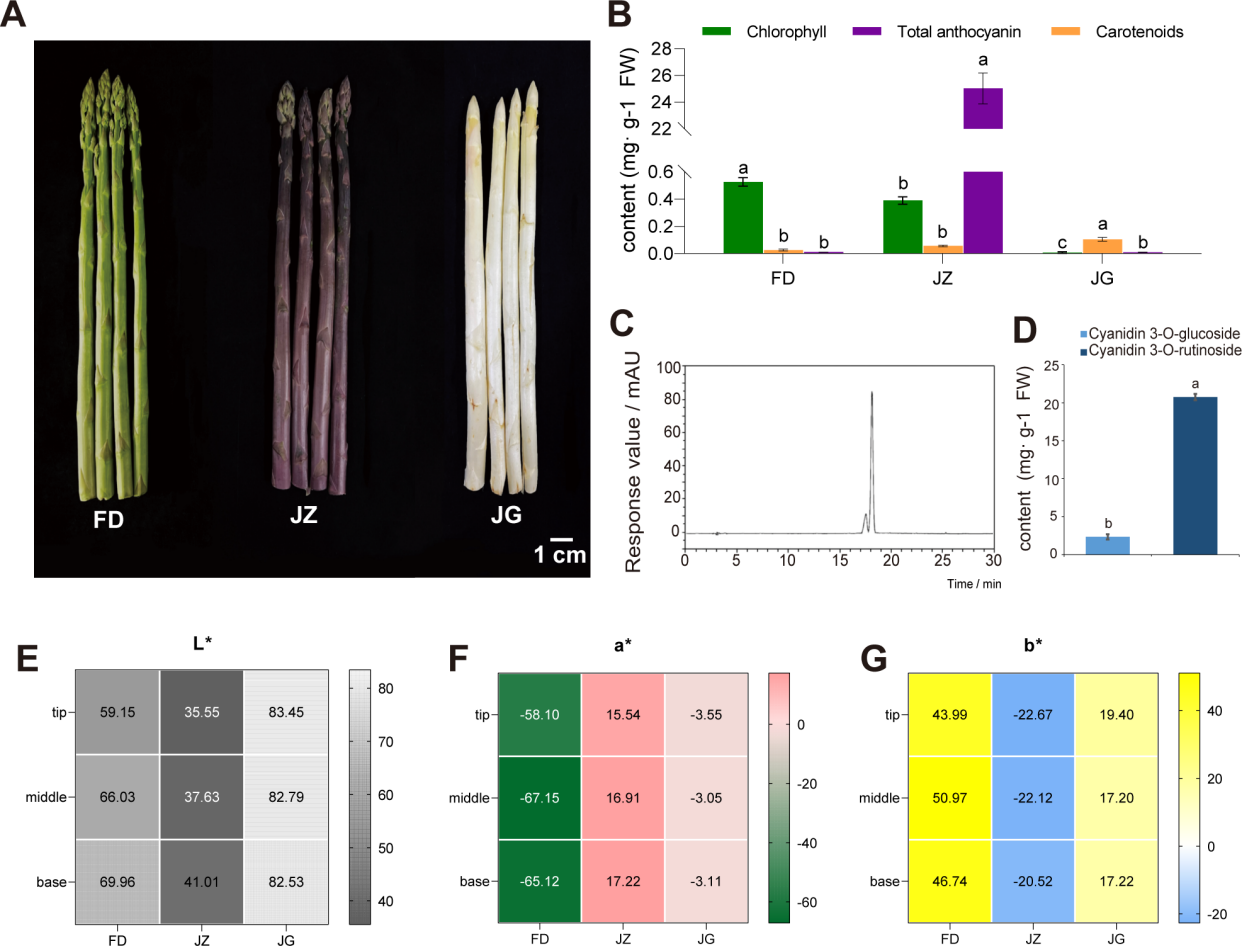
Three different colors of asparagus and analysis of differences. **(A)** Phenotypic images of three kinds of asparagus, from left to right: FD, JZ, and JG. **(B)** Chlorophyll, carotenoid and total anthocyanin content of the three asparagus varieties. **(C)** HPLC analysis of anthocyanins in JZ. **(D)** Bar graph of the anthocyanin glycoside content in JZ. **(E)** Heatmap of L* distribution. **(F)** Heatmap of a* distribution. **(G)** Heatmap of b* distribution. T-test at the significance level (*p* < 0.05), with different letters (a, b, and c) indicating significant differences and the same letters indicating non-significant differences.

The anthocyanin accumulation in plants can affect the coloration of plant tissues. The differences in appearance among the three asparagus were primarily in the epidermis of the tender stems (**Figure 1A**). FD was green, and some of its scales were darker. JZ appeared bright purple, and JG appeared pale yellowish-white. To further quantify the color differences, colorimeter was used to measure the L*, a*, and b* values of the tip, middle, and base sections of the asparagus. The results, shown in **Figures 1E–G**, reveal stable values that correspond closely to the visual appearance of the colors in each section. **Figure 1B** shows that only FD and JZ contain chlorophyll and they all contained less carotenoid than chlorophyll. However, carotenoid was significantly more abundant in JG than the other two varieties, possibly due to its yellowish color. The total anthocyanin content of JZ was significantly higher than that of FD and JG, further supporting the appearance characteristic of JZ showing purple color. HPLC was used to detect anthocyanin aglycones in the epidermis of three types of asparagus tender stems to precisely analyze the specific components of asparagus anthocyanins. These results revealed that the HPLC chromatogram of JZ primarily consisted of two peaks (**Figure 1C**). The retention times for cyanidin 3-glucoside and cyanidin 3-rutinoside were 17.3 and 18.0 min, respectively. The cyanidin 3-rutinoside content was particularly high, 8.86 times that of cyanidin 3-glucoside (**Figure 1D**). In contrast, white and green asparagus did not exhibit a characteristic peak, indicating a low anthocyanin content, which is almost negligible.

### 3.2 Transcriptome sequencing and analysis of DEGs

The cDNA libraries were constructed and sequenced using the Illumina NovaSeq 6000 platform to further investigate transcriptome changes in the epidermis of asparagus stems of varying colors. Nine cDNA libraries were created comprising three asparagus colors and three biological repeats. A total of 400,509,822 raw reads were generated. A total of 387,268,684 high-quality clean reads were obtained by screening. On average, 80.66% of the clean reads were mapped to the asparagus genome (**Supplementary Table S3**). Principal component (PCA) and correlation analysis (**Supplementary Figures S1A, B**) indicated the high reproducibility of the three biological replicates within each group and significant differences between the groups.

**Figure 2:**
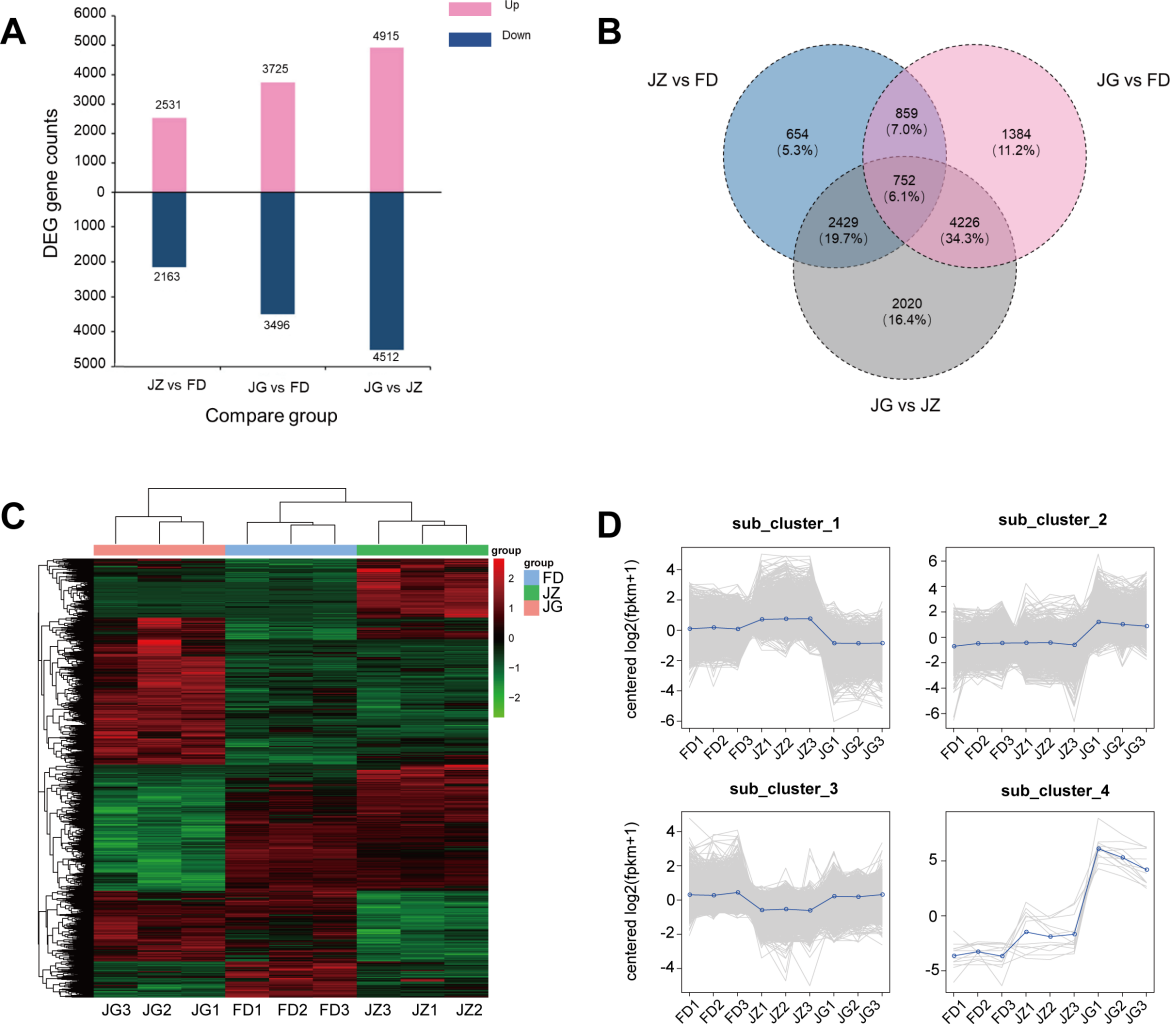
Characterization of the distribution of DEGs in the three different comparison combinations. **(A)** Number of DEGs in two different comparisons. Blue indicates downregulated DEGs, and pink indicates upregulated DEGs. **(B)** Wayne plots of prevalent and unique expressions in pairwise comparisons, presenting the number of DEGs versus proportion. **(C)** Hierarchical clustering of DEGs in all samples. **(D)** K-means clustering of gene expression trends. The grey line represents the relative corrected expression level of each gene, and the blue line represents the mean expression level of each gene.

**Figure 2A** shown the number of DEGs for each combination of comparisons. Among the three pairs of comparison groups, the JG versus JZ comparison group exhibited the highest number of DEGs, with 4,512 downregulated and 4,915 upregulated genes. Furthermore, the Wayne plot illustrates that the greatest number of DEGs was identified between the JG versus FD comparison group and the JG versus JZ comparison group, amounting to 4,978 (40.4%) of the total (**Figure 2B**). A total of 752 genes were expressed in all three comparison groups, while 2,020 genes were expressed exclusively in the JG versus JZ comparison group. Further analysis revealed that these DEGs are involved in multiple biological pathways, particularly those related to anthocyanin biosynthesis, including phenylalanine metabolism, phenylpropanoid biosynthesis, and flavonoid biosynthesis. These findings are in strong agreement with the metabolic pathways identified in the subsequent annotation analysis, providing valuable insights into the molecular mechanisms underlying anthocyanin biosynthesis. A hierarchical clustering heatmap of 12,324 DEGs was constructed using the normalized FPKM Z-score values (**Figure 2C**). The hierarchical clustering heatmap demonstrated comparable expression patterns for JZ and FD genes but distinct expression patterns for JG genes. K-means clustering identified four subclasses with varying mean expressions (**Figure 2D**). Cluster 1 was the most abundant cluster. JZ exhibited high and low expression levels in clusters 1 and 3, respectively. In contrast, JG exhibited a high expression level of 3,930 genes in cluster 2 and 12 genes in cluster 4, indicating significant alterations in expression patterns.

### 3.3 DEGs annotation analysis

**Figure 3:**
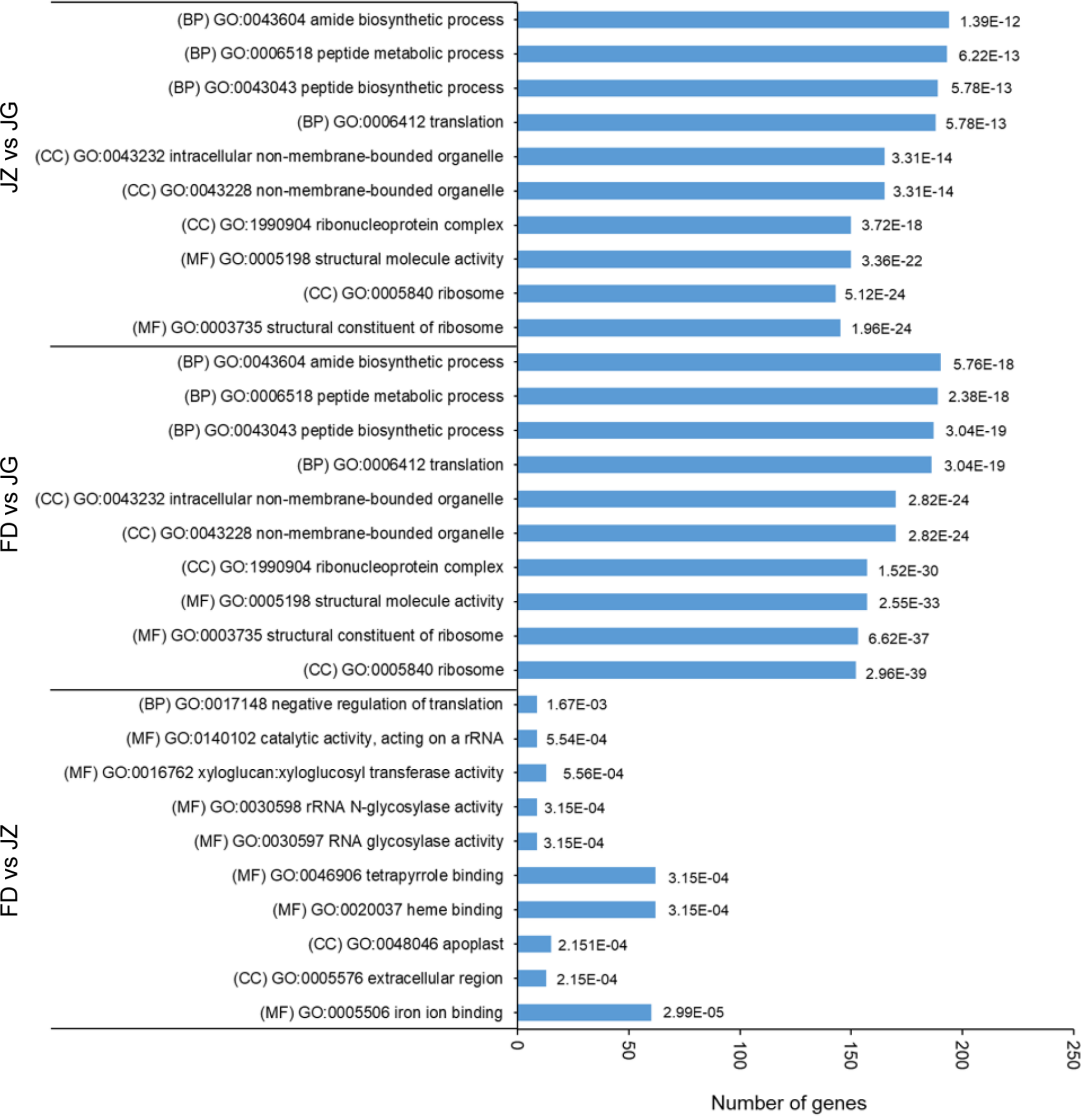
GO for the top 10 enrichment items in each pair comparison (*p* ≤ 0.05). The bar chart presents the error-finding rate values. BP, biological processes; MF, molecular function; CC, cell component.

Transcriptome profiles of diverse germplasms were analyzed to investigate the dynamic expression patterns of specific genes associated with anthocyanin accumulation. GO analysis was used to categorize the annotations of DEGs. The top 10 significantly enriched annotated terms were selected, including three main GO categories: cellular components (CC), biological processes (BP), and molecular functions (MF) (**Figure 3**). Most DEGs were enriched in MF, followed by CC and BP. These genes are associated with anthocyanin accumulation and may play pivotal roles in MF. Regarding BP, the most abundant subcategories were peptide biosynthetic process (GO:0043043), peptide metabolic process (GO:0006518), and amide biosynthetic process (GO:0043604). The number of genes enriched in the amide biosynthetic process (GO:0043604) was highest in the FD versus JG and JZ versus JG comparison groups. In contrast, FD and JZ were primarily enriched in iron ion binding (GO:0005506), heme binding (GO:0020037), and tetrapyrrole binding (GO:0046906) in MF. These findings indicated that the color difference observed in asparagus stems was associated with anthocyanins and photosynthetic pigments.

**Figure 4:**
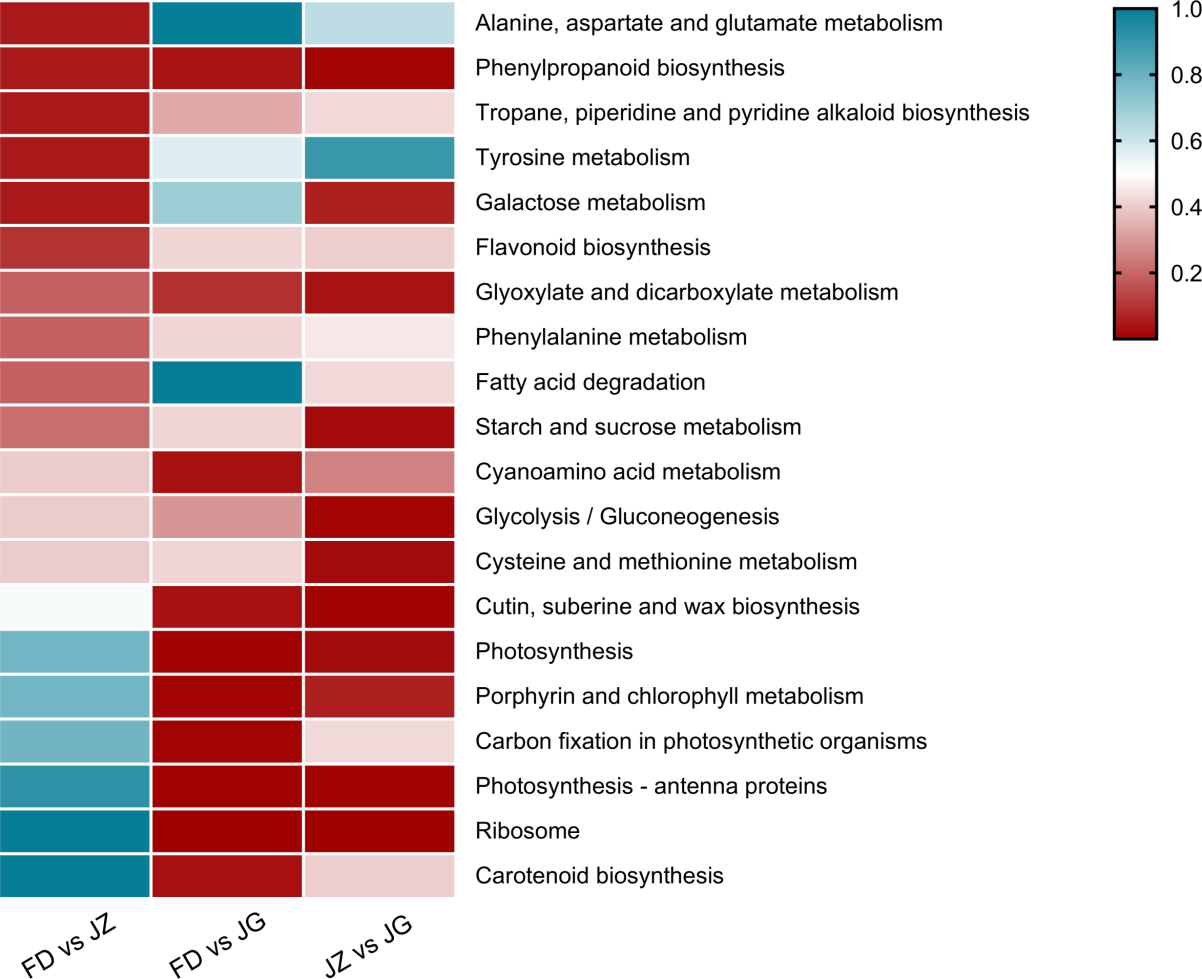
DEG enrichment analysis of comparative pairs using KEGG. The graph is based on the q-value of enrichment in the pathway. The redder the color, the more obvious the enrichment. The top 20 enrichment paths are listed below. Significant differential genes were determined according to error discovery rate ≤ 0.05 and (|log2 ratio| ≥ 1).

Subsequently, DEGs were mapped to the KEGG database to study metabolic pathways. The numbers of DEGs in the three comparison groups were 1,512, 2,737, and 3,319, respectively. These genes were assigned to 116, 117, and 117 metabolic pathways, respectively. Twenty metabolic pathways with significant expression were selected to generate heatmaps (**Figure 4**). Some DEGs were mapped to anthocyanin biosynthesis-related pathways, including phenylpropane biosynthesis, phenylalanine metabolism, and flavonoid biosynthesis. The phenylpropane biosynthesis pathway was significantly enriched for all three combinations. The flavonoid biosynthesis and phenylalanine metabolism pathways exhibited significant enrichment exclusively in the FD versus JZ comparison groups. However, some chlorophyll-related metabolic pathways, including photosynthesis-antenna proteins, porphyrin, chlorophyll metabolism, and photosynthesis, were significantly enriched in the FD versus JG and JZ versus JG comparison groups. Furthermore, the carotenoid biosynthesis pathway was identified as being significantly enriched in the FD versus JG comparison group. Thus, these differences in pigment metabolic pathways may underlie purple asparagus formation.

### 3.4 Analysis of key structural genes related to anthocyanin biosynthesis

**Figure 5:**
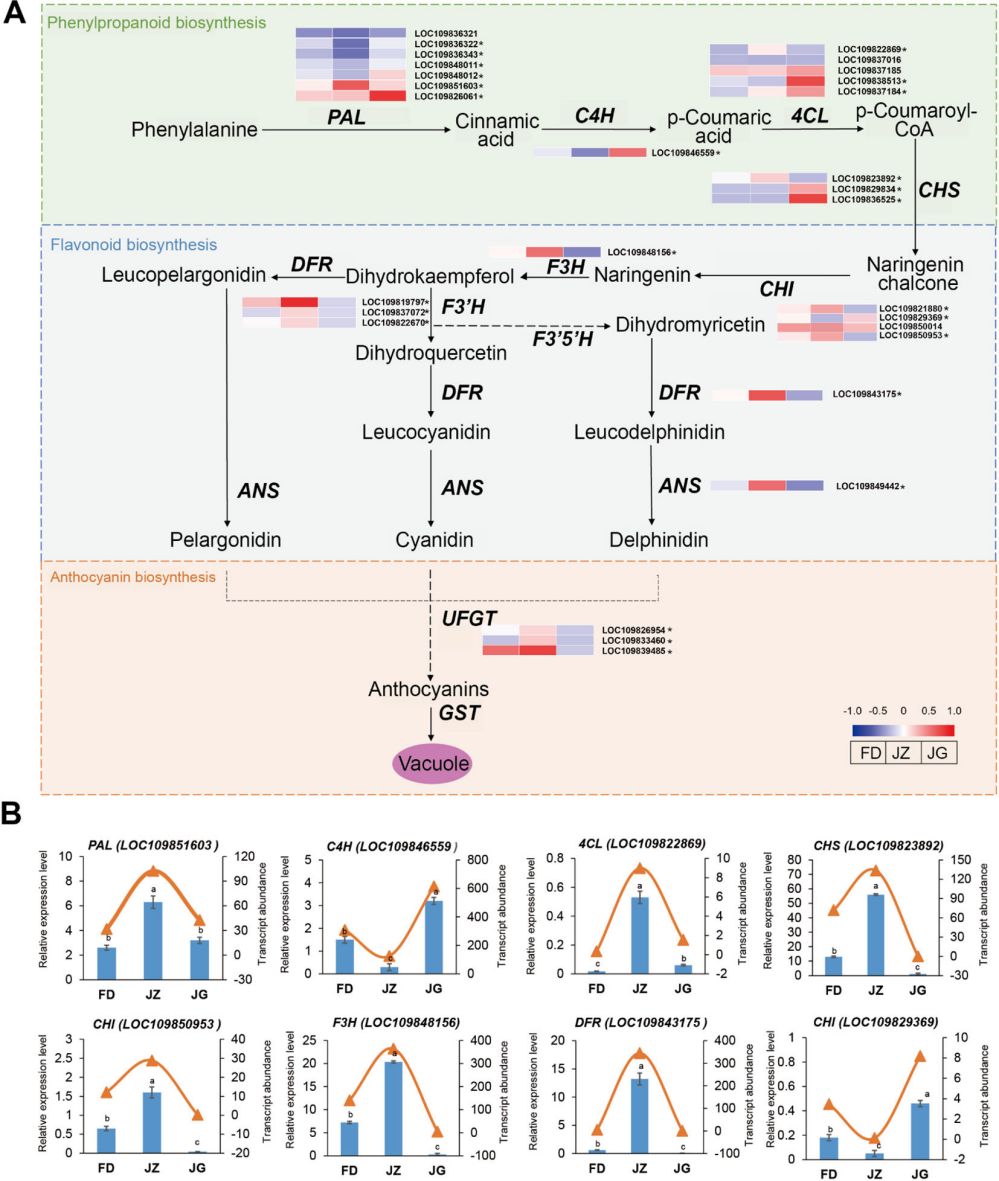
**(A)** Schematic diagram of anthocyanin synthesis pathway and expression changes in related structural genes in asparagus. Red (upregulated) and blue (downregulated) in the heatmap represent the gene expression trends. * indicates that the gene is DEG. **(B)** RT-qPCR analysis of the key pathway genes. The bar graph represents the relative expression of genes in RT-qPCR, and the triangular curve represents the RNA sequencing data. Each column represents the mean ± standard deviation (SD). T-test at the significance level (*p* < 0.05), with different letters (a, b, and c) indicating significant differences and the same letters indicating non-significant differences.

A schematic diagram of the anthocyanin synthesis pathway in asparagus was constructed based on the key enzymes involved in anthocyanin biosynthesis related to the color formation (**Figure 5A**). The asparagus transcriptome revealed 29 genes involved in anthocyanin biosynthesis. These genes are involved in the phenylpropanoid, flavonoid, and anthocyanin biosynthesis pathways and encode 10 enzymes (**Supplementary Table S4**). Among these biosynthesis pathways, phenylpropanoid biosynthesis pathway serves as the common starting point for various metabolic pathways. It is capable of providing precursor substances. Flavonoid biosynthesis pathway can furnish a carbon framework for the synthesis of anthocyanins, and anthocyanin biosynthesis pathway is able to transform colorless anthocyanins into colored ones. The expression levels of the 12 structural genes encoding phenylalanine ammonia-lyase (PAL) and p-coumaroyl CoA ligase (4CL) were either upregulated or downregulated across all three asparagus varieties. This complex and irregular expression pattern may be due to their involvement in the shared flavonoid metabolic pathway. Four genes encoding CHS and CHI were highly expressed in JZ. The elevated expression of these genes may be associated with flavonoid accumulation. The expression levels of genes encoding F3H, flavanone 3’-hydroxylase (F3’H), DFR, and ANS were significantly elevated in JZ. These genes play pivotal roles in determining the structural and chromatic characteristics of anthocyanins. Therefore, this may be related to the accelerated accumulation of anthocyanins during this phase. The anthocyanidin 3-O-glucosyltransferase expression was significantly reduced in JG. The diverse modifying effects observed at this stage altered asparagus coloration. This may be associated with the absence of anthocyanins that form a stable structure.

To ascertain the veracity of the RNA-seq data, eight genes involved in anthocyanin synthesis were randomly selected for RT-qPCR (**Figure 5B**). The phenylpropane biosynthesis pathway genes *PAL* (LOC109851603) and *4CL* (LOC109822869) exhibited the highest expression in JZ and were significantly upregulated, with one- and eight-fold increases, respectively, compared to JG. The expression of cinnamate 4-hydroxylase (*C4H*) (LOC109846559) was highest in JG and lowest in JZ. Flavonoid biosynthesis pathway genes, including *CHS* (LOC109823892), *F3H* (LOC109848156), and *DFR* (LOC109843175), exhibited the highest expression levels in JZ. The lowest expression of *CHI* (LOC109850953) was observed in JG, while the highest expression was observed in *CHI* (LOC109829369). These findings align with the transcriptomic sequencing results.

### 3.5 Differential expression transcription factor analysis

**Figure 6:**
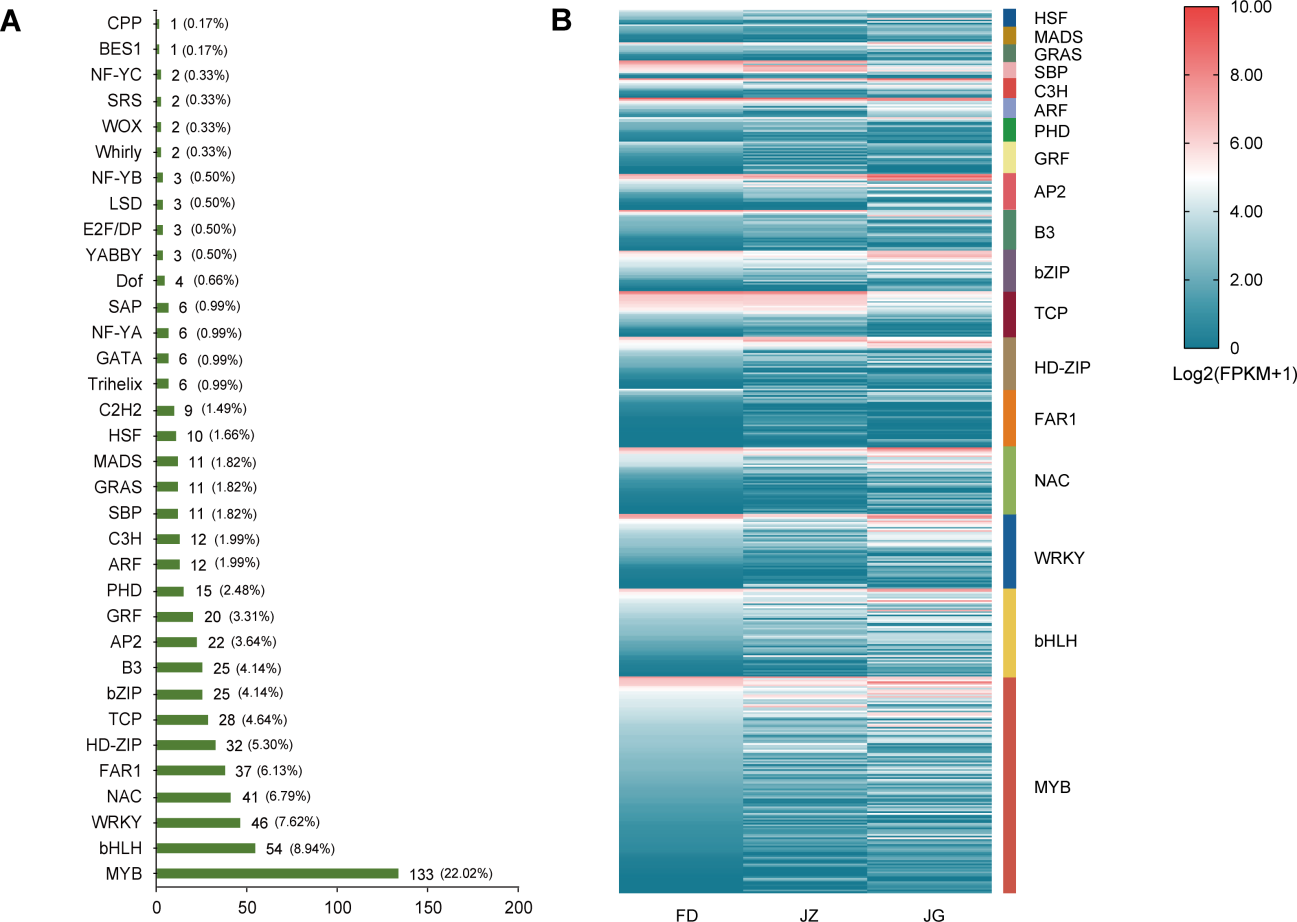
Differentially expressed transcription factors in asparagus. **(A)** The transcription factor family, the corresponding number of transcription factors, and the percentage of each family. **(B)** Specific expression of the transcription factors. Blue to red indicates expression from low to high. Expression was calculated using log2 (FPKM+1), and the heatmap depicts the transcription factor families of more than 10 family members.

The TFs of DEGs were analyzed, and nomenclature was assigned using the Plant Transcription Factor Database. A total of 604 DEGs were identified as TFs and classified into 34 distinct families, including MYB, bHLH, WRKY, NAC, FAR1, and others (**Figure 6A**). The three families with the highest numbers of genes containing TFs were MYB (n = 133), bHLH (n = 54), and WRKY (n = 46). The heatmap of the gene expression levels for each TF family member demonstrated that MYB, bHLH, and WRKY were expressed in all treatments (**Figure 6B**). In the JZ versus FD comparison group, 15 MYB, six bHLH, and four WRKY genes exhibited increased expression. In the JZ versus JG comparison group, 16 MYB, 10 bHLH, and four WRKY genes exhibited increased expression. These differences in expression indicate that TFs regulate anthocyanin synthesis via multiple mechanisms.

### 3.6 Cloning of *AoMYB114* and expression patterns in different asparagus varieties

**Figure 7:**
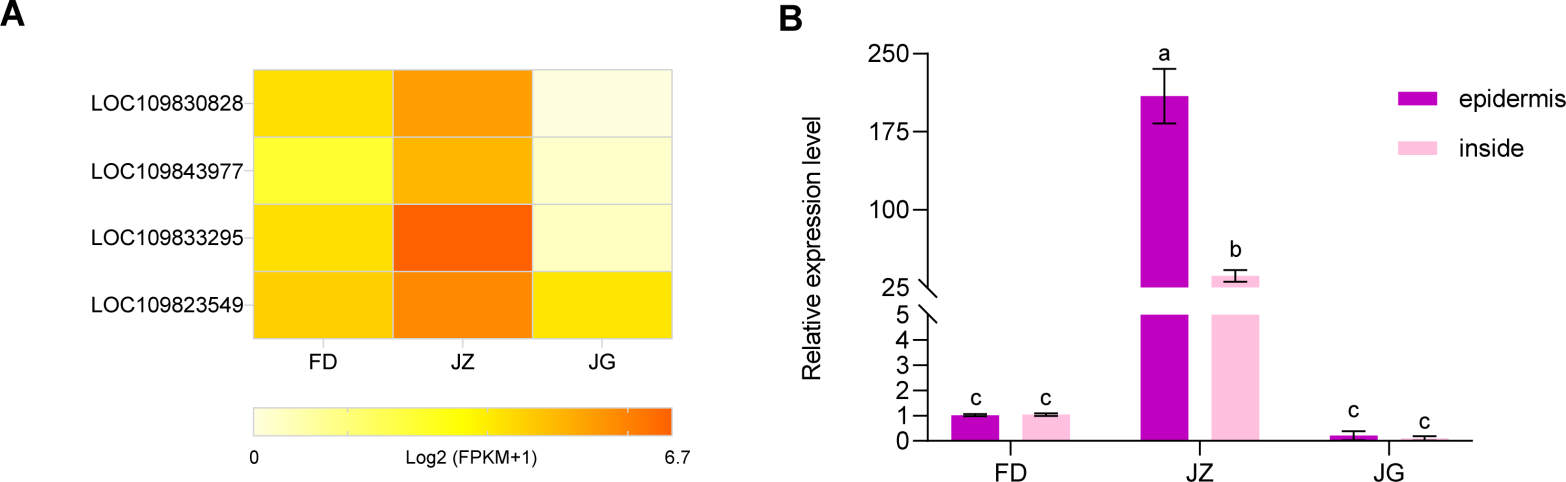
**(A)** Heatmap of the expression abundance of the four MYB transcription factors in different-colored asparagus transcriptomes. Data are represented as Log2 (FPKM+1). **(B)** Expression analysis of the asparagus *AoMYB114* gene in different asparagus and different parts of the asparagus. T-test at the significance level (*p* < 0.05), with different letters (a, b, and c) indicating significant differences and the same letters indicating non-significant differences.

MYB transcription factors have many members and the most intricate functions, and they are the most significant transcriptional regulatory factors in plant metabolic networks. Based on the analysis of the existing transcripts of major genes regulating epidermal anthocyanin biosynthesis, four MYB transcription factors were screened (**Figure 7A**). The expression levels of the three asparagus varieties were significantly different, with JZ displaying the highest expression levels. In contrast, expression levels were relatively lower in FD and JG. In particular, LOC109833295 (*AoMYB114*) exhibited minimal expression in the white epidermis and the highest expression in the purple epidermis. Therefore, based on transcriptome data, the transcription factor *AoMYB114*, related to anthocyanin synthesis in asparagus, was preliminarily identified. It was cloned from the JZ (**Supplementary Figure S2A**), and sequencing results demonstrated that *AoMYB114* contained an open reading frame of 711 bp, encoding 236 amino acids (**Supplementary Figure S2B**). Homology analysis of *AoMYB114* (**Supplementary Figures S2C, D**) revealed a high degree of homology with R2R3-MYB transcription factors from other species. For example, the following sequences were identified: *Hippeastrum* hybrid cultivar (WHL30745.1), *Muscari armeniacum* (AVD68967.1), *Allium cepa* (AQP25671.1), *Phoenix dactylifera* (UHT46070.1), *Freesia* hybrid cultivar (QJW70307.1), *Magnolia liliiflora* (AHJ60260.1), *Rubroshorea leprosula* (GKV47092. 1), *Magnolia sinica* (XP_058071391.1) and *Elaeis guineensis* (XP_010931211.1). They all possessed conserved structural domains of R2 and R3 and contained a bHLH-binding motif within the R3 domain.

The expression pattern of *AoMYB114* in tender asparagus stems was studied using RT-qPCR (**Figure 7B**). In JZ, *AoMYB114* relative expression level was significantly higher in the epidermis than in the interior. Furthermore, its expression was low in FD and JZ. These findings suggest that *AoMYB114* is specifically expressed in tissues with a high anthocyanin content.

### 3.7 Overexpression of the *AoMYB114* in *Arabidopsis thaliana*

**Figure 8:**
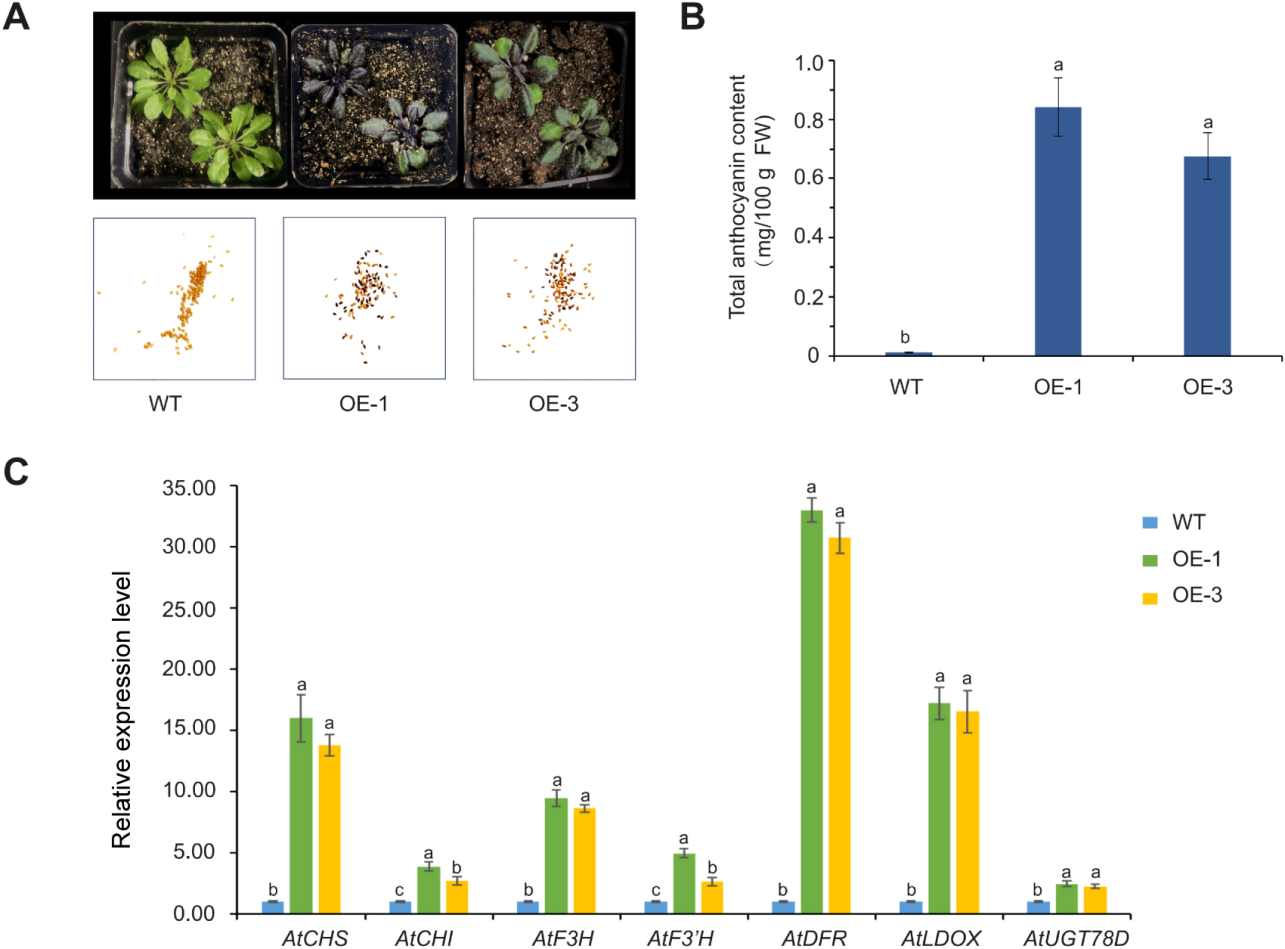
*AoMYB114* was overexpressed in *Arabidopsis*. **(A)** The phenotypes of transgenic *Arabidopsis* and WT *Arabidopsis*, from left to right, are WT, transgenic *Arabidopsis* OE-1, and transgenic *Arabidopsis* OE-3, with T3 generation *Arabidopsis* seedlings on top and T1 generation *Arabidopsis* seeds on the bottom. **(B)** Comparison of total anthocyanin content between transgenic and WT *Arabidopsis*. The error line represents the standard deviation of the data (SD, n = 3), and lowercase letters represent significant differences (*p* < 0.05). **(C)** Relative expression levels of structural genes of the anthocyanin synthesis pathway in transgenic and WT *Arabidopsis*. Different letters (a, b, and c) indicating significant differences and the same letters indicating non-significant differences.

A plant overexpression vector, pCAMBIA1301-AoMYB114, was constructed to ascertain the function of *AoMYB114*. Genetic transformation of *Arabidopsis thaliana* was performed using the Agrobacterium GV3101 mediated floral dip method. Four lines were isolated from the 1/2 MS agar plates. The transgenic *Arabidopsis* lines (OE-1 and OE-3) were selected for further experiments. PCR amplification of *AoMYB114* was conducted using cDNA derived from wild-type (WT) and transgenic *Arabidopsis*. PCR products of approximately 700 bp were detected in the two transgenic lines, whereas no PCR products were detected in the WT. Transgenic *Arabidopsis* plants exhibited GUS activity (**Supplementary Figure S3**). As shown in **Figure 8A**, the majority of T1 generation *Arabidopsis* seeds exhibit a purple color, indicating that the gene is effectively expressed in T1 seeds. Moreover, the same purple phenotype was observed in T3 generation *Arabidopsis* seedlings, suggesting that the gene has demonstrated genetic stability in this generation. The total anthocyanin content of the two transgenic *Arabidopsis* was significantly higher than that of the WT (**Figure 8B**). The total anthocyanin content of the OE-1 line reached 0.87 mg/100 g FW. The anthocyanin content in plants is usually related to the expression of structural genes. The effect of *AoMYB114* gene overexpression on structural gene expression of the anthocyanin biosynthesis pathway in transgenic *Arabidopsis* was examined by RT-qPCR. The expression levels of all structural genes in the transgenic lines were higher than those in WT (**Figure 8C**). In particular, *AtDFR, AtLDOX*, and *AtCHS* gene expression reached the highest level in OE-1 and significantly increased by 32, 16, and 15 times compared to WT. These differences suggest that *AoMYB114* is overexpressed by enhancing the structural gene expression for anthocyanin biosynthesis.

## 4 Discussion

Commercial asparagus can be classified into three categories based on its color: white, green, and purple. Studies on anthocyanin content in asparagus have revealed that purple varieties have the highest anthocyanin concentration (Slatnar et al., 2018). This study also corroborates the finding that the anthocyanin content of purple asparagus tender stems was significantly higher than that of non-purple asparagus. In contrast to other flavonoids, anthocyanins were highly unstable in their free state. Anthocyanins typically undergo glycosylation to form stable anthocyanidin glycosides to enhance stability. Sakaguchi et al. (2008) isolated anthocyanins from purple asparagus and identified them as cyanidin 3-[3″-(O-β-d-glucopyranosyl)-6″-(O-α-l-rhamnopyranosyl)-O-β-d-glucopyranoside] and cyanidin 3-rutinoside. Li et al. (2012) identified three principal anthocyanin glycosides in the purple asparagus extract: cyanidin-3-glucoside-(rhamnopyranosyl)-5-glucoside, cyanidin-3-rutinoside, and peonidin-3-rutinoside. In this study, the presence of two anthocyanidin glycosides, cornflower-3-O-glucoside, and cornflower-3-O-rutinoside, in purple asparagus tender stems was confirmed using HPLC analysis. The concentration of cornflower-3-O-rutinoside was 8.86 times greater than that of cornflower-3-O-glucoside. In contrast, the JG variety of white asparagus and the FD variety of green asparagus did not exhibit characteristic peaks, and the anthocyanin content was exceedingly low. The principal anthocyanin constituent of JZ was identified as cornflower-3-O-glucoside, consistent with the findings of Slatnar et al. (2018). This provides a foundation for further investigation into the mechanisms underlying the purple pigmentation of asparagus.

Anthocyanin biosynthesis is an important branch of the flavonoid pathway. Although the types and accumulation patterns of anthocyanins differ among different species, the synthesis process is the same. A series of structural genes regulate these genes. In this study, 29 structural genes involving the encoding of 10 enzymes were identified. *CHS* and *CHI* play important roles in flavonoid accumulation; however, the anthocyanin biosynthesis pathway is non-specific. For example, the anthocyanin content in *Clivia miniata* is closely related to *CHS* expression (Viljoen et al., 2013). However, in the three peony varieties (red-purple, white, and yellow), *CHI* was highly expressed throughout the developmental stage (Zhao et al., 2012). The *TfCHI* expression level initially decreased and then increased (Yuan et al., 2013). In this study, seven structural genes encoded CHS and CHI. The *CHI* (LOC109850014) gene was highly expressed in all three colors of asparagus, and only four were highly expressed in purple asparagus. The high expression of these genes may be involved in the early stages of flavonoid biosynthesis, which provides precursors for anthocyanin synthesis. *F3H* can catalyze the conversion of naringenin to dihydroflavone and biosynthesize flavanols and anthocyanins. The *F3H* downregulation in strawberries decreased the flavanol and anthocyanin contents (Jiang et al., 2012). The *F3H* expression level in mulberry fruits rich in anthocyanins was positively correlated with anthocyanin accumulation (Dai et al., 2022). DEGs encoding F3H and F3’H expression levels were also upregulated in purple asparagus. DFR and ANS are key enzymes in late synthesis. When activated by transcription factors, the *StANS* gene can promote anthocyanin synthesis in potato tubers (Zhang et al., 2020). In this study, DEGs encoding DFR and ANS were significantly upregulated in purple asparagus. These structural genes are key genes that regulate the color of asparagus tender stems. The flavonoid biosynthesis pathway is the key metabolic pathway involved in the purple formation of asparagus tender stems. The phenylpropane metabolic pathway is the common starting point for various metabolic pathways, including flavonoids, lignin, and tannins (Dong and Lin, 2021). Since *p*-coumaroyl-CoA is the central metabolite that connects anthocyanin and lignin biosynthesis, it’s the compound that both secondary metabolic pathways compete for. The DEG encoding C4H was significantly upregulated in white asparagus. This could be due to the metabolism moving to other branches.

Structural genes directly influence anthocyanin biosynthesis by encoding key enzymes. Transcription factors precisely regulate the expression pattern and intensity of these structural genes, thereby controlling the temporal and spatial expression changes in anthocyanins. This intricate regulation forms the basis for pigment formation in plants, which is crucial for plant adaptation to environmental changes and for the coloration of plant organs (Yan et al., 2021). Currently, MYB, bHLH, and WD40 are the major transcription factors that regulate anthocyanin biosynthesis (Ramsay and Glover, 2005). These transcription factors regulate the expression of key enzymes in the anthocyanin biosynthetic pathway either cooperatively or independently. The cooperative action of these transcription factors forms a stable transcriptional regulatory network, while their positive or negative regulatory roles vary depending on environmental changes and physiological state (Jun et al., 2015). In the transcriptome analysis of this study, a total of 604 transcription factors were identified, belonging to 34 transcription factor families, with the MYB and bHLH families having the highest number of genes. This finding further emphasizes the importance of MYB and bHLH families in plant anthocyanin biosynthesis. MYB transcription factors, in particular, play a central role in plant pigment synthesis due to their structural specificity and regulatory diversity. Transcription factors can also interact with structural genes to regulate the accumulation and distribution of anthocyanins. Some studies have demonstrated that *GMYB10* is involved in anthocyanin synthesis in the leaves and flowers of *Gerbera hybrida*. This is primarily accomplished through interaction with bHLH factor *GMYC1* to activate DFR (Elomaa et al., 2003). Meanwhile, some MYB transcription factors exert negative regulatory effects. For example, *AtMYBL2*, a negative regulator, plays a role in inhibiting anthocyanin biosynthesis (Matsui et al., 2008). These phenomena indicate that MYB transcription factors may function within complex regulatory networks in different plant varieties or tissues, adapting to various environmental conditions and growth states.

R2R3-MYB transcription factors play an important role in anthocyanin biosynthesis. They are generally considered more specific (Zhang et al., 2014). These transcription factors show significant spatiotemporal expression differences across various developmental stages and tissues of plants. For example, *MaMybA* had the highest expression level, primarily in the S3 stage of flower development, whereas the relative expression level in roots, bulbs, and leaves was low (Chen et al., 2019). Similarly, *LhMYB12-Lat* contributed to the formation of splashes on the petals of *Lilium* spp. but did not cause complete pigmentation of the perianth and spots (Yamagishi et al., 2014). These studies highlight the high spatiotemporal specificity of different R2R3-MYB transcription factors in various plant species and developmental stages, which is an essential feature of their role in regulating anthocyanin biosynthesis. In this study, *AoMYB114* was specifically expressed in asparagus tissues with high anthocyanin content. This result suggests that *AoMYB114* may play an important regulatory role in anthocyanin biosynthesis in asparagus. More importantly, ectopic *AoMYB114* overexpression in *Arabidopsis* activates structural genes associated with anthocyanin biosynthesis. It significantly promoted *AtCHS, AtDFR*, and *AtLODX* gene expression, thereby increasing anthocyanin accumulation in *Arabidopsis*. This finding indicates that *AoMYB114* not only plays an essential regulatory role in asparagus but can also effectively regulate anthocyanin biosynthesis in an exogenous expression system, further demonstrating the universality and regulatory potential of the *AoMYB114* transcription factor in plant pigment synthesis.

## 5 Conclusion

This study demonstrated that anthocyanin accumulation primarily influenced the coloration of JZ tender stems. The purple coloration of the tender asparagus stems was predominantly due to cyanidin 3-glucoside and cyanidin 3-rutinoside. The structural genes associated with anthocyanin biosynthesis exhibited heightened expression in tender purple stems. Thus, the anthocyanin biosynthesis pathway was activated. RT-qPCR confirmed this differential expression. Based on asparagus transcriptome sequencing results, *AoMYB114* was identified in JZ. This gene was specifically expressed in tissues with a high anthocyanin content and exhibits high homology with MYB transcription factors related to anthocyanin synthesis in various plants. Moreover, *AoMYB114* overexpression induces anthocyanin biosynthesis in *Arabidopsis*. Consequently, *AoMYB114* positively regulates anthocyanin biosynthesis and may play a pivotal role in developing purple epidermal traits in asparagus. These findings are important in optimizing asparagus quality and breeding high-anthocyanin plant varieties.

## Data Availability

The datasets presented in this study are deposited in NCBI, with the accession number PRJNA1220106.
