# Supplementary material for: AoMYB114 transcription factor regulates anthocyanin biosynthesis in the epidermis of tender asparagus stems
Source: Front Plant Sci. 2025 Feb 18;16:1531574. doi: 10.3389/fpls.2025.1531574 (PMC11876374; doi:10.3389/fpls.2025.1531574)
Supplement: Supplementary file 1 [file DataSheet1.pdf]

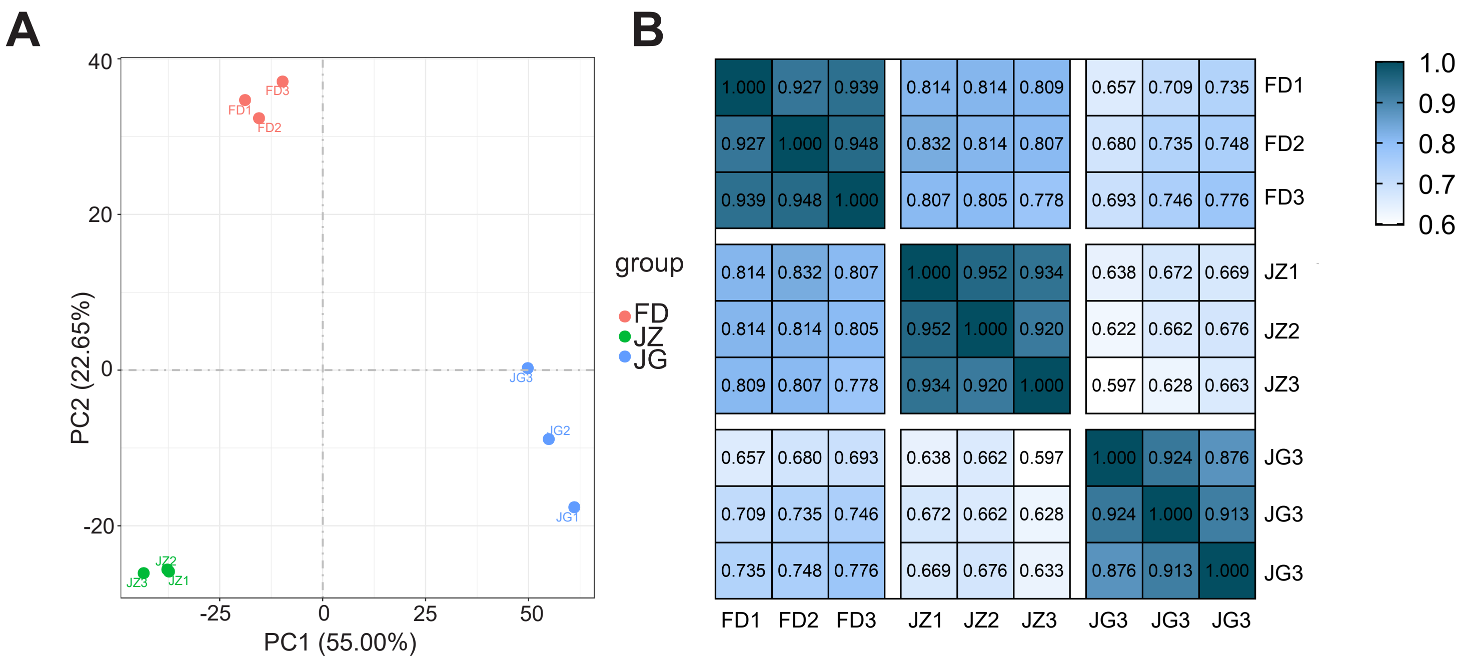

Figure S1. (A) Principal Component Analysis (PCA) plot for each group, where 1, 2, and 3 represent three biological replicates (the same below). **(B)** Pearson correlation coefficient heatmap of gene expression between different samples.
