# Supplementary material for: AoMYB114 transcription factor regulates anthocyanin biosynthesis in the epidermis of tender asparagus stems
Source: Front Plant Sci. 2025 Feb 18;16:1531574. doi: 10.3389/fpls.2025.1531574 (PMC11876374; doi:10.3389/fpls.2025.1531574)
Supplement: Supplementary file 2 [file DataSheet2.pdf]

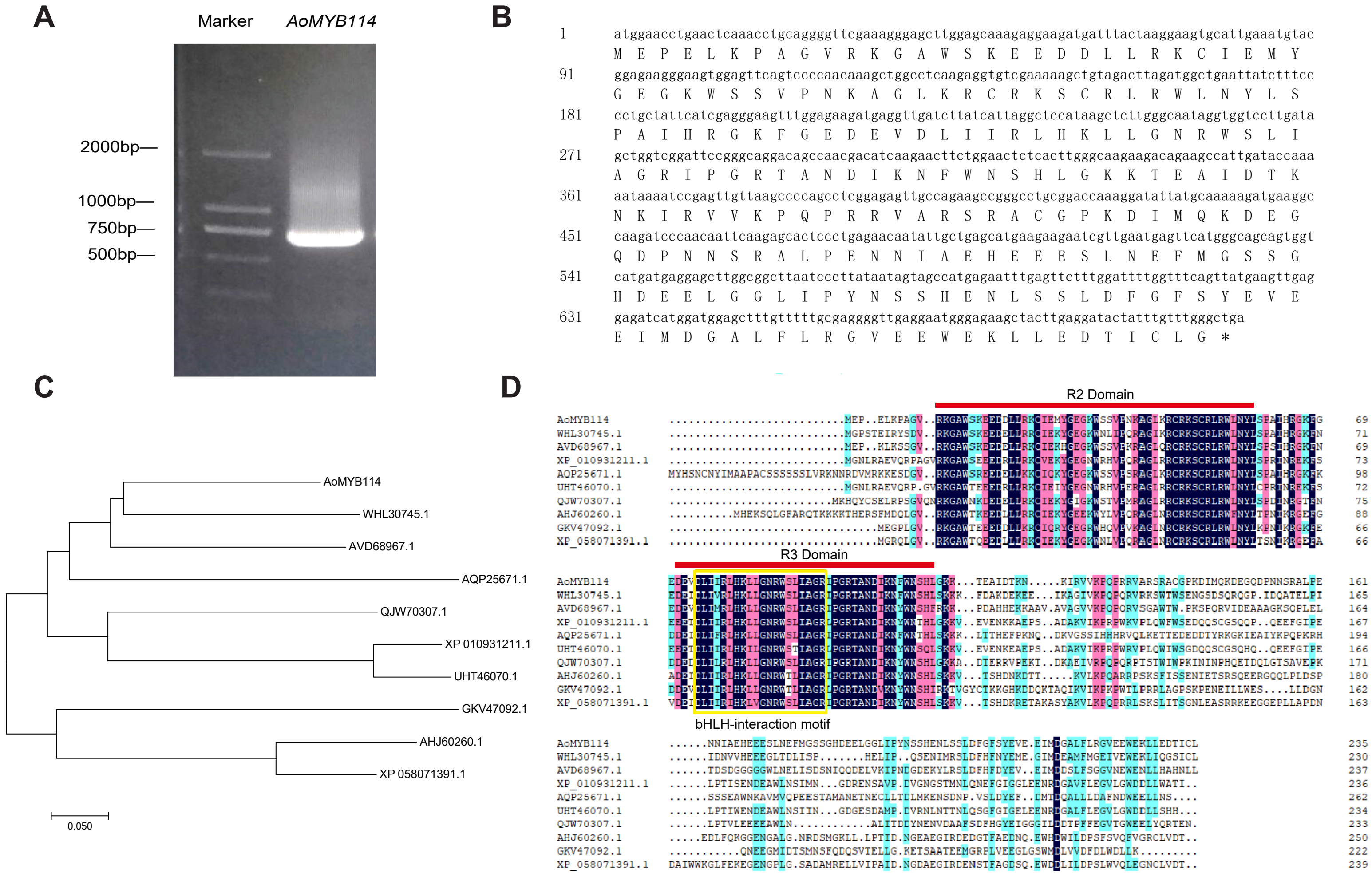

Figure S2. (A) Amplification of *AoMYB114*. **(B)** The nucleotide sequence of Asparagus *AoMYB114* and the amino acid sequence of the encoded protein. **(C)** Evolutionary tree of Asparagus *AoMYB114* and R2R3-MYB transcription factors in other species. **(D)** Sequence alignment of Asparagus *AoMYB114* and other R2R3-MYB transcription factor amino acids. The red line represents the R2 domain and R3 domain, and the yellow rectangle represents the bHLH-interaction motif.
