# Supplementary material for: AoMYB114 transcription factor regulates anthocyanin biosynthesis in the epidermis of tender asparagus stems
Source: Front Plant Sci. 2025 Feb 18;16:1531574. doi: 10.3389/fpls.2025.1531574 (PMC11876374; doi:10.3389/fpls.2025.1531574)
Supplement: Supplementary file 3 [file DataSheet3.pdf]

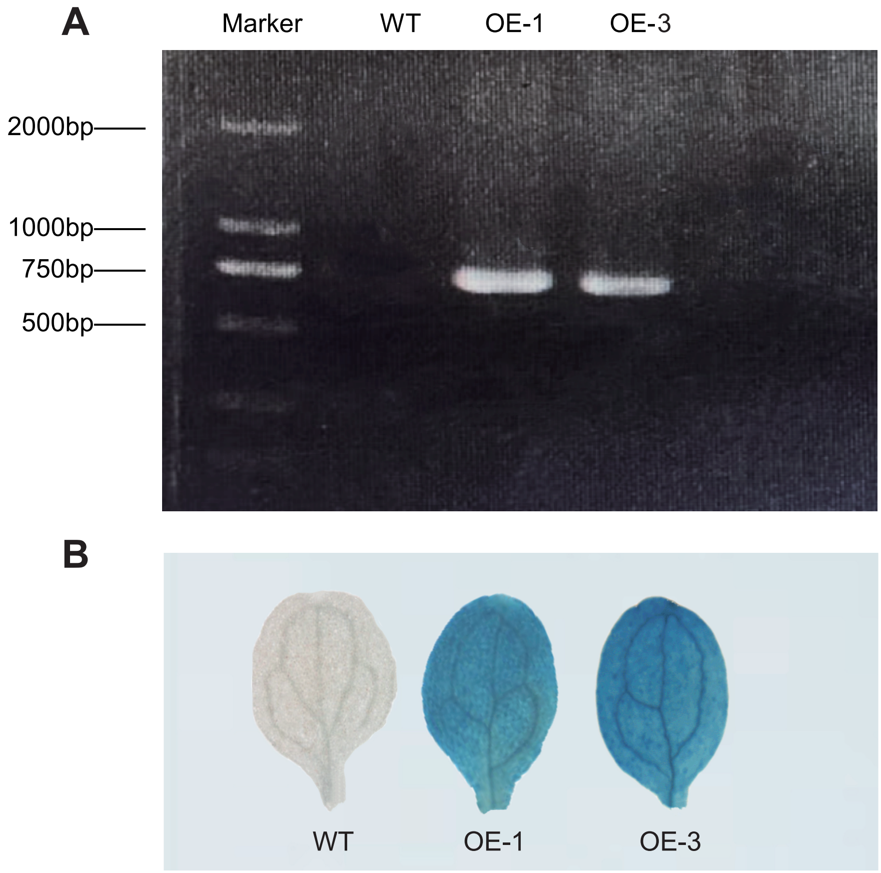

Figure S3. (A) PCR amplification experiments of *AoMYB114* in wild-type and transgenic Arabidopsis. (B) The GUS staining of Arabidopsis thaliana, from left to right, consists of wild-type, transgenic Arabidopsis OE-1, and OE-3.
